# Development and experimental verification of C-arm camera shooting locator

**DOI:** 10.1038/s41598-022-26286-9

**Published:** 2022-12-23

**Authors:** Jun Yang, Lin Yang, Tae Gyong Jon, Zejun Fang, Peng Jin, Zhenghua Hong, Fei Ye, Jiawen Wang

**Affiliations:** 1 Sanmen People’s Hospital, Taizhou, 317100 Zhejiang China; 2 School of Forensic Medicine, Guizhou Medical University, Guiyang, 550004 Guizhou China; 3 First Affiliated Hospital of Zhejiang University School of Medicine, Hangzhou, 310000 Zhejiang China; 4 Taizhou Hospital of Zhejiang Province, Taizhou, 317000 Zhejiang China

**Keywords:** Techniques and instrumentation, Orthopaedics, Medical imaging

## Abstract

This study aimed to develop a self-made C-arm camera shooting locator and verify its accuracy and advantages. A total of 60 physicians and nurses from the Surgical System of Sanmen People’s Hospital, Zhejiang Province, China, were randomly selected as filming operators. The C-arm machine with a self-made locator and a C-arm machine without a locator were used to measure the center of the circular plate. The iron nails were used to shoot. The distance between the iron nail and the center point of the circular display area on display was defined as the shooting deviation. When it was less than 3 cm, the shooting was stopped. The number of shots, total shooting time, and first-shot deviation in the C-arm camera shooting groups with and without the locator were statistically analyzed, and the advantages and disadvantages of the two were compared. The average number of shots, average total shooting time, and average first-shot deviation of the C-arm camera using the locator were significantly better than those in the group without the locator, and the differences were statistically significant. When the shooting distance (X) was equal to 30 cm and the shooting angle (Y) was equal to 0°, the average number of shots, average total shooting time, and average first-shot deviation were optimal. The C-arm camera shooting locator can improve the shooting accuracy of the C-arm camera and effectively reduce the number of shots and total shooting time. Hence, it can be applied in clinical and surgical practice.

## Introduction

**Figure 1:**
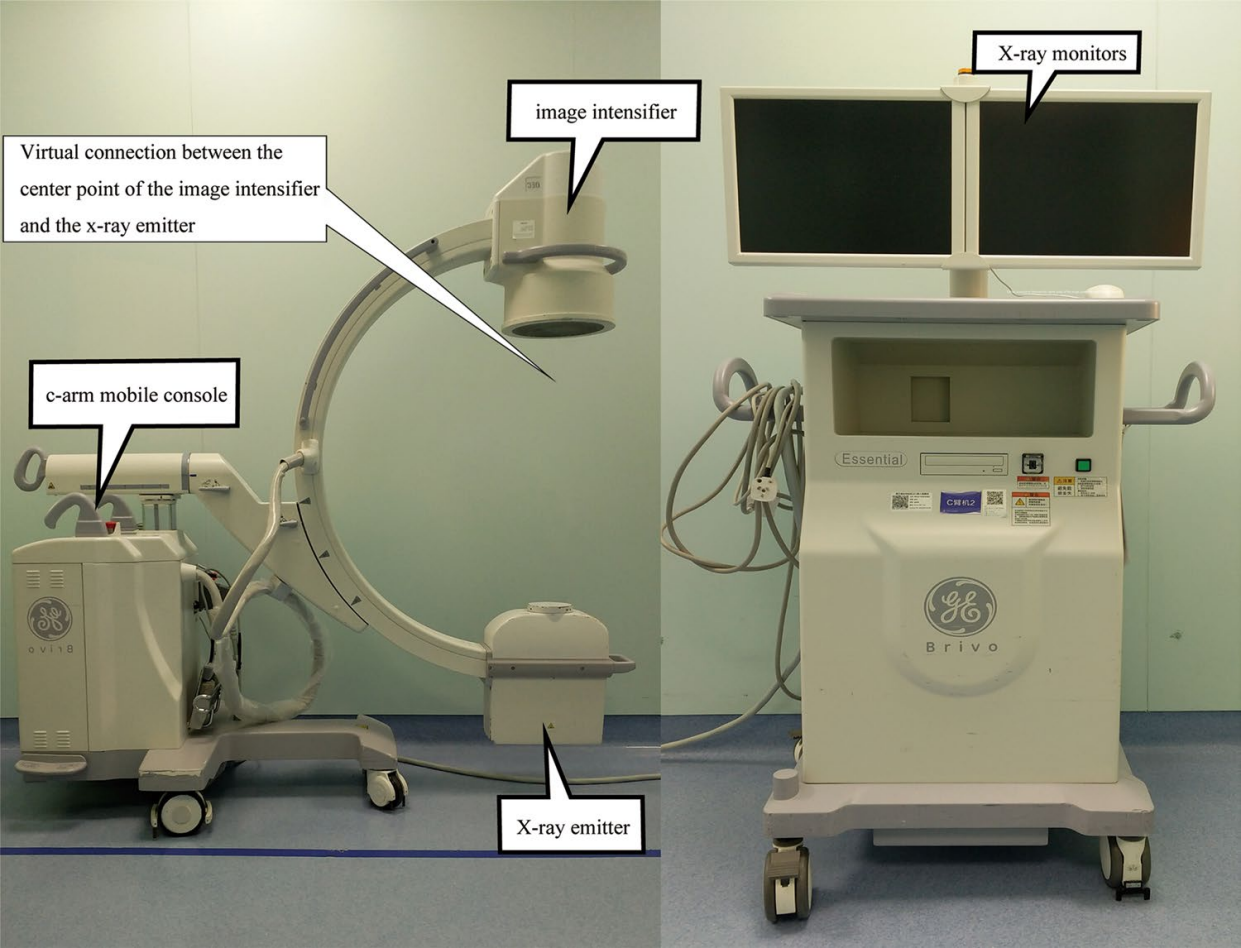
C-arm machine, model: Siemens PLX7000.

A C-arm x-ray machine (referred to as C-arm machine,model: Siemens PLX7000, as shown in Fig. 1) is a mobile x-ray machine that integrates light, machine, and image processing technologies^1^, which is used for real-time dynamic imaging in surgery. It is a commonly used surgical auxiliary tool in clinical orthopedics. The main uses include assisting in fracture reduction and fixation during orthopedic surgery, assisting in implanting a pacemaker, assisting in extracting foreign bodies from the body, assisting in part of angiography and interventional operations, cooperating with an ozone machine to treat pain, cooperating with small needle-knife treatment, assisting gynecological tubal guidance surgery, and so forth^2–6^. It has the advantages of low infection risk, small footprint, and easy movement. It has been widely used in orthopedics, general surgery, gynecology, and other departments. In order to obtain high quality images, it is necessary to adjust the line between the center point of the image intensifier of the C-arm machine and the center point of the x-ray emitter to pass exactly through the center point of the subject to achieve pre-alignment^7^. However, the current clinical C-arm machines basically do not have a pre-alignment function and requiring multiple adjustments and repeated shooting to obtain satisfactory images^1,7–13,20–22,26,28^.

Previous studies have shown that 80% of shooting procedures require repositioning of the C-arm machine^11^. Ionizing radiation can cause damage to a variety of human tissues. Also, excessive x-ray exposure can cause tumors, hematopoietic diseases, cataracts, cardiovascular diseases, and neurodegenerative diseases^14–18^. Multiple filming inevitably increases the x-ray exposure time of patients and medical workers, and also increases the ionizing radiation damage to both doctors and patients. Therefore, in 2010, the US Food and Drug Administration issued a white paper advocating to reduce exposure to unnecessary medical imaging^3^. In addition, repeated radiographic operations prolong the operation time and increase the risk of surgical bleeding, postoperative infection, and thrombosis, leading to serious consequences for patients^1,7–9^.

In order to reduce the time of X-ray exposure of patients and medical staff, the author's team independently developed a C-arm camera accurate shooting locator and verified the shooting efficiency and accuracy of the locator at different shooting distances and different shooting angles using experimental methods.

## Results

**Figure 2:**
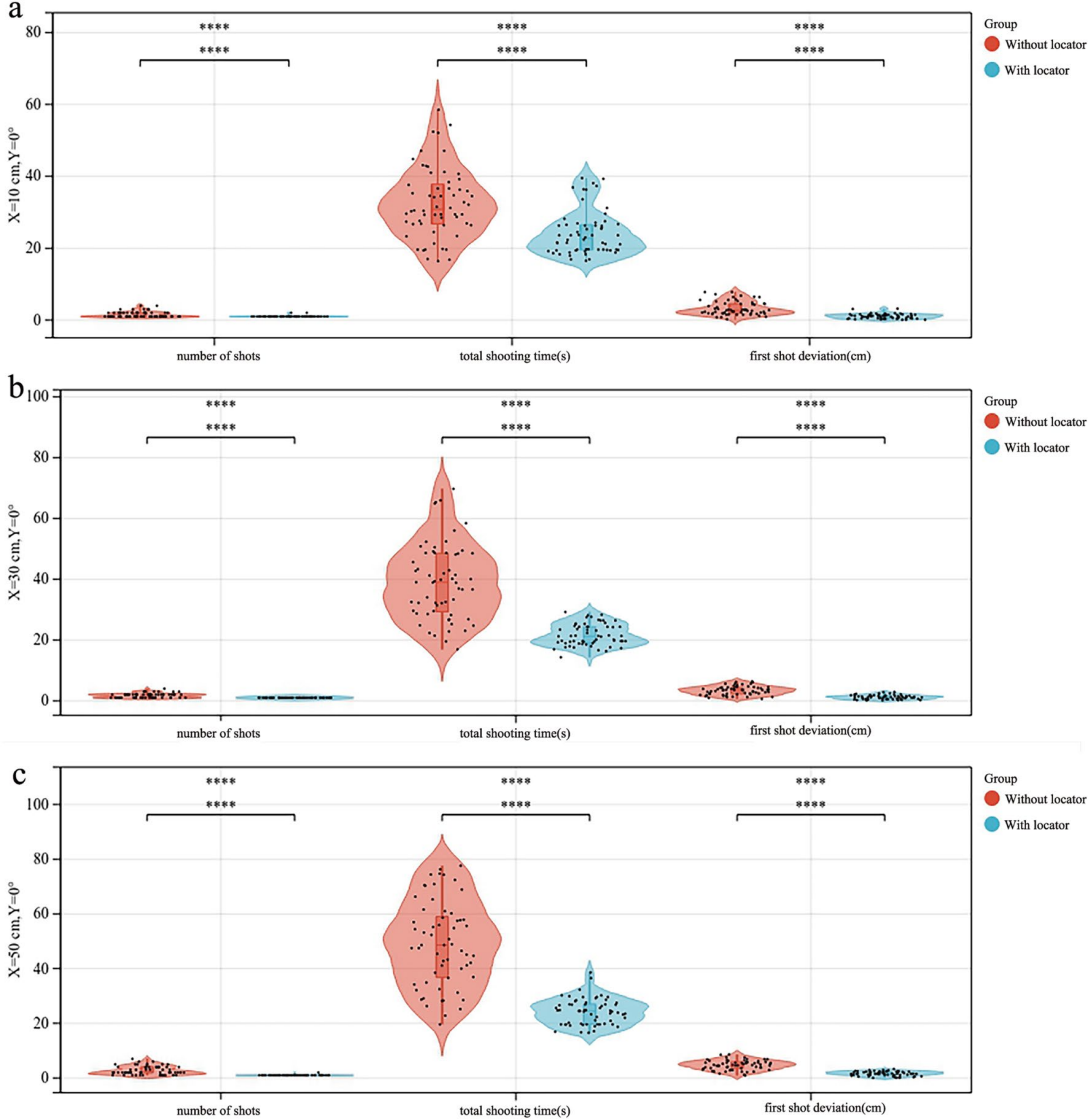
Comparison of the number of shots, total shooting time, and first-shot deviation between the control and experimental groups. (**a**) when *X* = 10 cm and *Y* = 0°, (**b**) when *X* = 30 cm and *Y* = 0°, (**c**) when *X* = 50 cm and *Y* = 0°.

**Figure 3:**
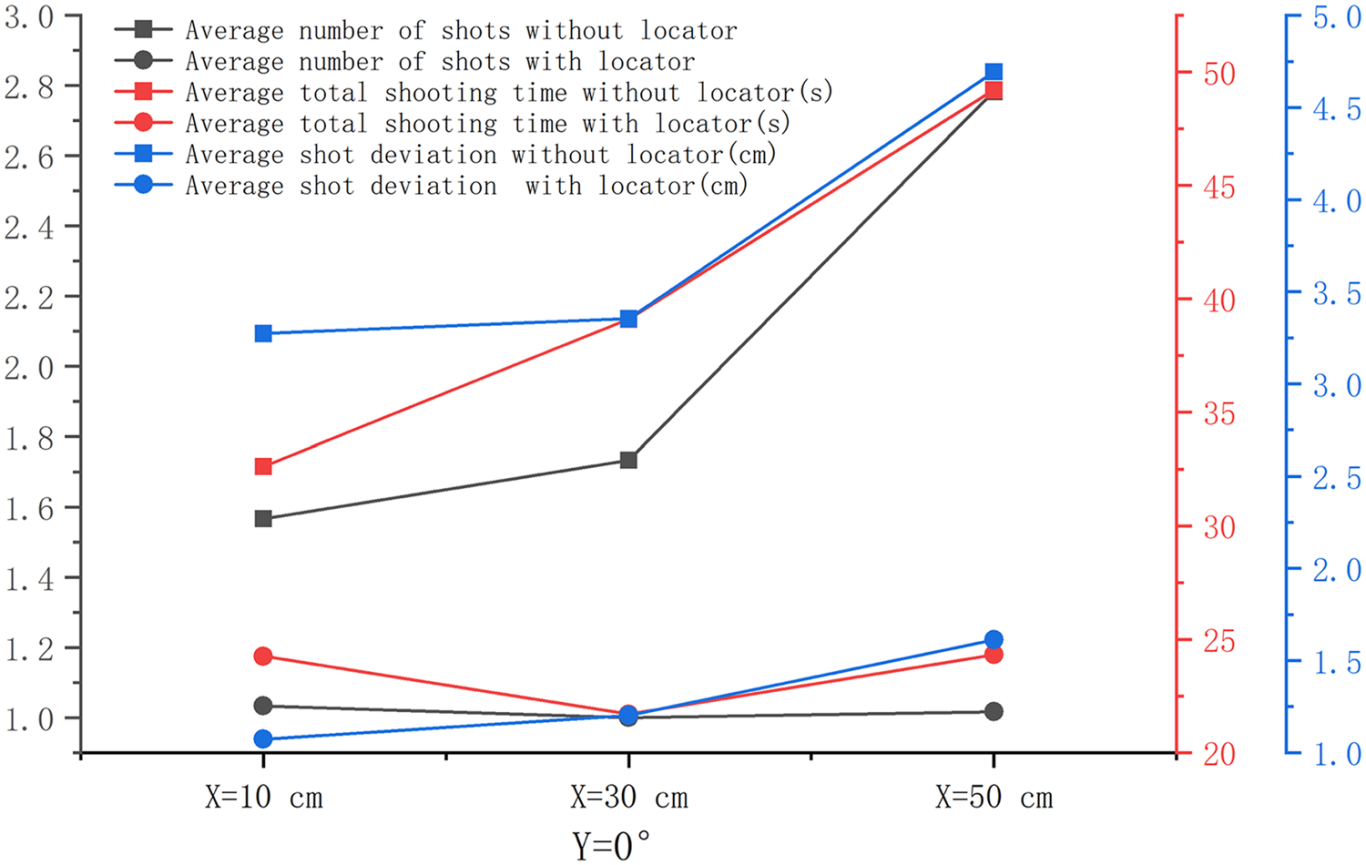
*Y* = 0° and *X* = 10 cm, 30 cm, and 50 cm. The change trend of the average number of shots, average total shooting time, and average first-shot deviation between the experimental and control groups.

When *Y* = 0°, the average number of shots, average total shooting time, and average first-shot deviation in the experimental group were significantly smaller than those in the control group, and the differences were statistically significant (*P* < 0.001). When *X* was equal to 50 cm, the difference was the largest (*P* < 0.001) (Fig. 2). When *Y* = 0°, the average total number of shots, the average total shooting time, and the average first-shot deviation in the experimental group showed no significant increasing trend with the increase in X, and the difference was not statistically significant (*P* > 0.05). In the control group, the average number of shots, average total time of shots, and average first-shot deviation showed a significant increasing trend, and the difference was statistically significant (*P* < 0.05), as shown in Fig. 3. The average total shooting time of the experimental group was the lowest at *X* = 30 cm. The average first-shot deviation in the control and experimental groups increased with the increase in the shooting distance (*P* < 0.05), but the average first-shot deviation in the experimental group was within the qualified range. Among these, when *X* = 30 cm and *Y* = 0°, the shooting time was the least and the shortest, and the shooting deviation was qualified.

**Figure 4:**
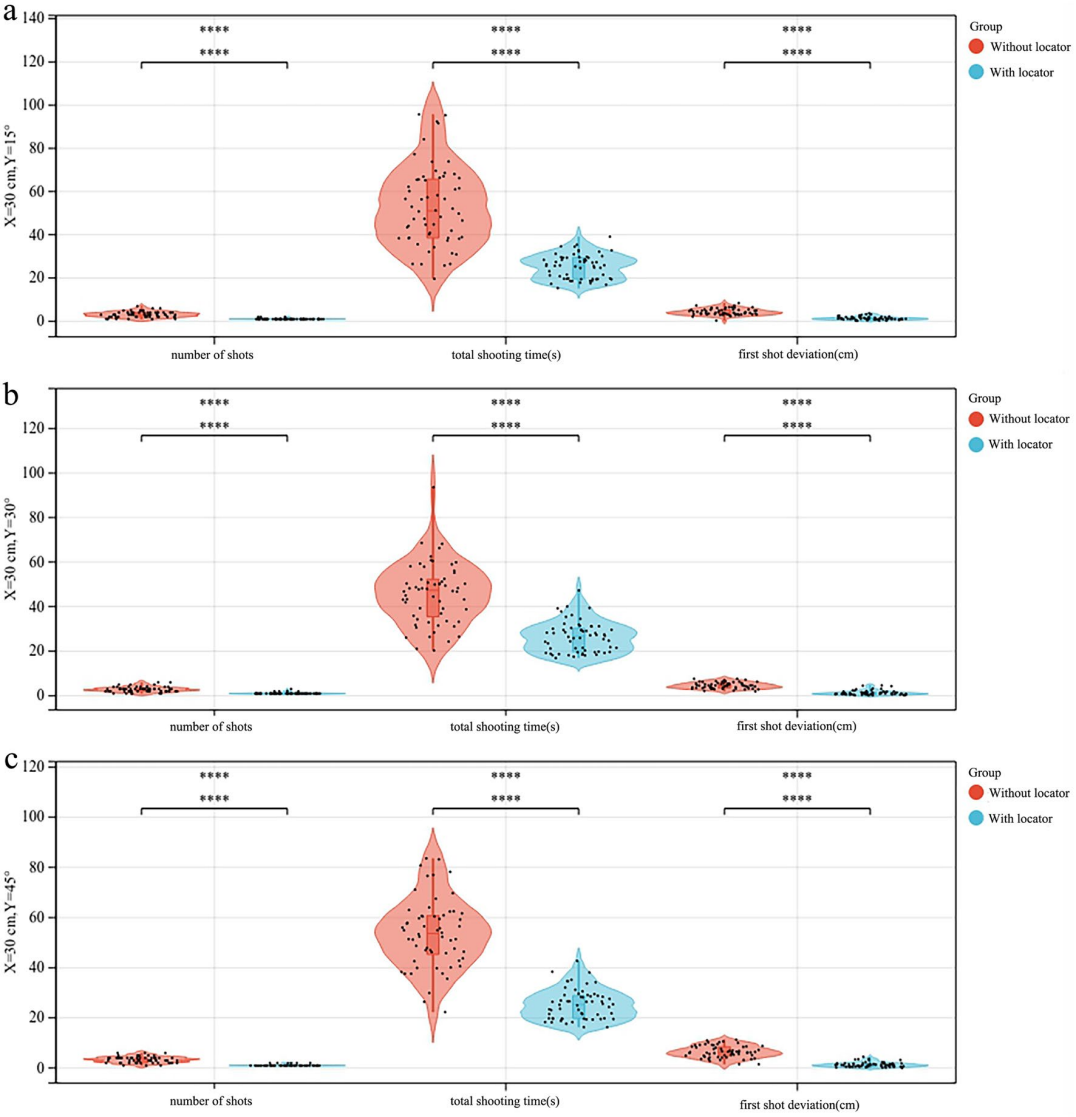
Comparison of the number of shots, total shooting time, and first-shot deviation between the control and experimental groups. (**a**) when *X* = 30 cm and *Y* = 15°, (**b**) when *X* = 30 cm and *Y* = 30°, (**c**) when *X* = 30 cm and *Y* = 45°.

**Figure 5:**
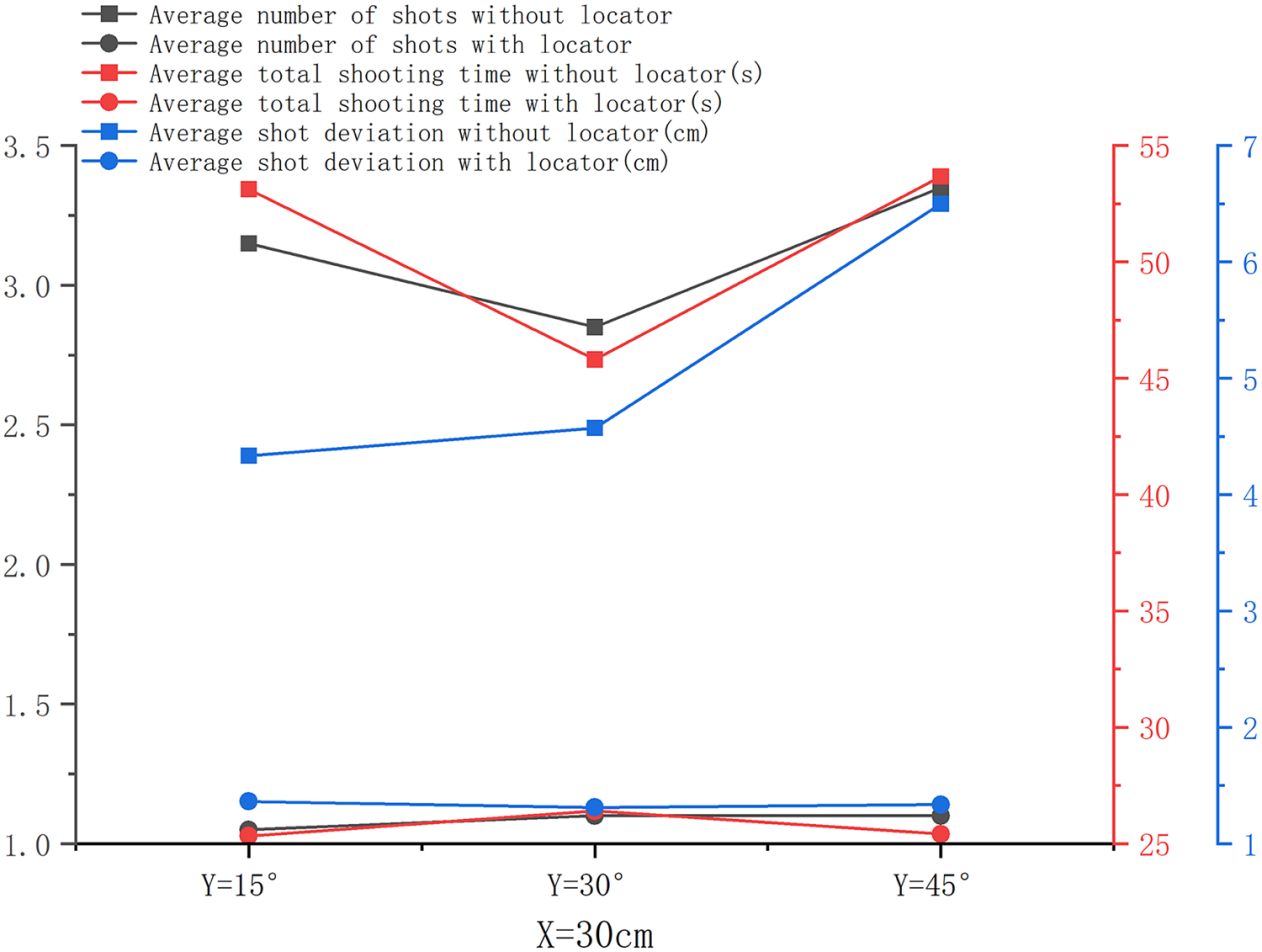
Change trends of the average number of shots, average total shooting time, and average first-shot deviation between the control and experimental groups when *X* = 30 cm and *Y* = 15°, 30°, and 45°.

When *X* = 30 cm and *Y* was the variable, the average number of shots, average total shooting time, and average first-shot deviation in the experimental group were significantly smaller than those in the control group. Especially when *Y* = 45°, the difference was the largest and statistically significant (*P* < 0.001), as shown in Fig. 4. When *X* = 30 cm, no significant difference was found in the average number of shots, average total shooting time, and average first-shot deviation in the experimental group with the increase in Y between the groups (*P* > 0.05). Also, the average number of shots in the control group increased with the change in *Y*, but the difference was not statistically significant (*P* > 0.05). Further, the average first-shot deviation increased with the increase in Y, and the difference was statistically significant (*P* < 0.05). The average total shooting time was the shortest when *Y* = 30°. The average total number of shots in the experimental group increased slightly with the increase in the shooting angle, but the difference was not statistically significant (*P* > 0.05). The change trend of the average total shooting time in the experimental group with Y was not statistically significant, and the maximum was at *Y* = 30°. The average first-shot deviation in the control group increased with the increase in *Y* (*P* < 0.05), and the average first-shot deviation in the experimental group had no significant change trend with the increase in *Y* and was minimum at *Y* = 30°, as shown in Fig. 5.

The aforementioned results showed that, regardless of the shooting height and angle, the number of shots, shooting time, and shooting deviation of the C-arm machine operator were greatly reduced in the experimental group, so as to meet the various requirements of the C-arm machine in actual clinical work. It could effectively reduce the radiation exposure time of both doctors and patients.

## Discussion

The experiments verified that the C-arm camera with the shooting locator developed by the author's research group had the following advantages: (1) It could achieve accurate shooting almost only once; (2) The average total shooting time was significantly lower than that in the control group; and (3) The average first-shot deviation was significantly lower than that in the control group. This showed that the locator could improve the performance of the C-arm machine.

At the same time, the experiment also found that when the locator was not used for shooting, the number of shots, the shooting time, and the first-shot deviation gradually increased with the increase in the distance between the C-arm image intensifier and the object. On the contrary, when shooting with a locator, the aforementioned indicators did not increase with the increase in shooting distance. However, when the shooting angle increased, the shape enclosed by the positioning laser point gradually changed from a circle to an ellipse, increasing the difficulty of the photographer in judging the center point and reducing the positioning effect of the object. Adding the center point positioning of the locator as a supplementary use was necessary in some special cases. The author's research group made a supplementary design for this defect and applied for a patent^19^.

C-arm machines have been widely used in surgery in various clinical departments, but in practical applications, the shooting accuracy is not high enough and multiple shootings are required to reduce shooting deviations, leading to excessive x-ray exposure for patients and medical staff. Taking more time leads to a decrease in the success rate of the surgery. A few scholars tried to improve the shooting accuracy of the C-arm machine by changing the shooting method: the " + " positioning method on the ground of the C-arm machine could effectively reduce the time of spinal surgery^20^. The effects of femoral trochanteric surgery were compared, revealing that using the perineal column as the reference material could significantly reduce the number of shots and effectively reduce the x-ray exposure time of patients and medical workers^21^. However, the aforementioned methods were limited only to a few cases in clinical application. Most scholars tried to improve shooting accuracy by improving the shooting equipment. For example, it was recently reported that the artificial x-ray system − generated simulated x-ray fluoroscopic images in real time to guide the shooting, but the total shooting time was not enough due to the problem of the simulated imaging lag in the system. Also, no shortening was noted^22^. Another example is the minimally invasive surgical positioning and navigation system of the C-arm machine. According to the intraoperative fluoroscopic image, a single laser point is instructed to point to the skin corresponding to the in vivo target, so as to achieve the body surface positioning of the in vivo target^23^. However, the positioning system is expensive and blocks the perspective range. It is mainly used to guide the removal of foreign bodies from the body, rather than to guide the C-arm machine for accurate shooting. In addition, the improvement in the aforementioned two shooting instruments involves more intermediate conversion links, many influencing factors, and high cost.

Laser lights are widely used in daily life and are easy to obtain and cost-effective. Hence, they have been used by scholars to improve the C-arm machine and hence the shooting accuracy^24–40^. However, they have two shortcomings in the following two aspects. First, in the research reports, the "center point" principle of the object is indicated by a single laser point or a cross line formed by multiple laser lines to guide the shooting. This center point is often the center point of the subject rather than the center point we need because the target is usually the bones of the human body, and the bones are located within the soft tissue coverage of the body surface. A certain gap exists between the center point of the bone and the center point of the object seen by the naked eye. The photographers unfamiliar with the anatomical structure mistakenly consider the center point of the subject as the center point of the shooting target, resulting in shooting deviation. The center point of the subject is also judged by estimation, with no clear mark of the center point of the subject. Harris et al. observed in a randomized prospective clinical study that laser localization did not reduce imaging time and radiation dose^24^. Second, the existing laser locator cannot meet the following two objectives at the same time: (1) continuously and effectively fixed on the C-arm, and (2) compatible with C-arms of various brands and specifications. Previous reports mentioned that laser light was pasted and fixed on the edge of the image intensifier for eccentric positioning^25^. Also, in some cases, the laser pointer was attached to the center point of the image intensifier during the operation, and the laser point was used to predict the shooting area, the center point^26^. The aforementioned methods had the following problems: (1) The laser pointer was not firmly fixed, and it could be deflected due to gravity or collision, resulting in the deviation of the pre-positioning. (2) The laser pointer blocked the x-ray, and the target image was incomplete. (3) The laser pointer must be sterilized and adjusted by the operator; also, a risk of infection exists. Xu Kewei et al.^27^ reported a laser dual positioning system scheme in which two lasers were installed on the image intensifier side and the transmitter side of the C-arm camera, and the " + " formed by the lasers was used for pre-positioning. However, the design did not mention how the laser light could be effectively fixed on different models of C-arm machines, and no clinical validation study was performed. Recently, Salih et al. customized a hoop locator especially for the OEC 9900 model in the GE brand^28^; however, it had the following problems: (1) The long antenna of the fixed ring was easy to collide with the surrounding objects and caused the fixed offset. (2) The exposed electronic components and lines easily caused the failure and affected the appearance. (3) The locator was not compatible with various types of C-arm machines.

In recent years, the author's research group has improved the aforementioned key issues and obtained 10 utility model patents^29–38^ and 1 domestic invention patent^39^. Some patents have been converted into prototypes. In addition, because the aforementioned new version of the locator was not compatible with some C-arm machines in a small number of hospitals, the author's group developed and designed a customized version of the locator as a supplement and obtained a Chinese utility model patent^40^. However, the C-arm camera shooting locator developed by the author's research group had features such as not-high aesthetics and a lack of human–computer interaction. The research group will make further upgrades and improvements to make the locator more complete and practical. In the future, we will explore the automatic locator under the condition of artificial intelligence technology to make it more intelligent and accurate.

## Conclusions

The locator used in this study can significantly reduce the number of shots, the shooting time, and the first-shot deviation. Hence, it is expected to minimize the ionizing radiation damage to both doctors and patients and reduce the incidence of surgical complications in patients.

## Methods

Sanmen People’s Hosipital approved the experiments, including any relevant details, all experiments were performed in accordance with relevant named guidelines and regulations and informed consent was obtained from all participants in this experiment.

### Model of C-arm machine and structure principle of the locator

**Figure 6:**
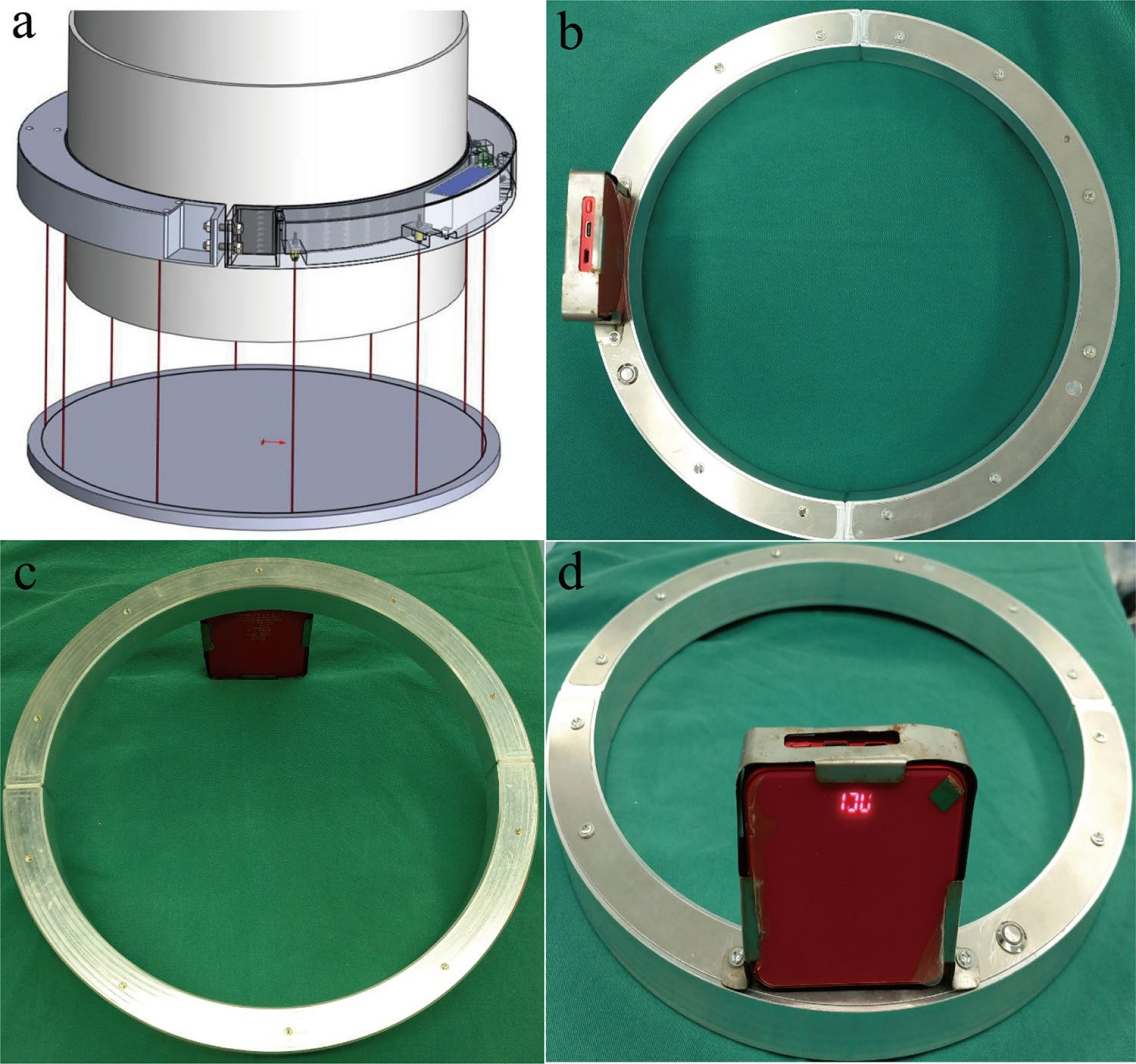
(**a**) Locator mounted on the image intensifier shooting 10 laser lines. (**b**) Top view of the locator. (**c**) Bottom view of the locator. (**d**) Side view of the locator.

The model of the C-arm machine used in this study was Brivo OEC 785 (Beijing General Electric Huatuo Medical Equipment Co., Ltd.). The self-made locator is shown in Fig. 6 (Taizhou Dingchuang Intelligent Technology Co., Ltd). It consisted of a "C"-shaped semi-circle A and a semi-circle B connected by bolts at both ends. The whole was a circular hoop, which could be fixed on the cylindrical image intensifier after tightening. A total of 10 laser lights, batteries, and remote control receivers were present in the housing of the locator. The 10 laser lights were equidistantly arranged and emitted 10 laser points to form a circle. The effect is shown in in Fig. 6a. The locator was provided with a main power switch, a power display, a charging hole, and a remote control receiver. The opening and closing of the laser light were controlled with a point remote control placed on the operating end of the C-arm.

### Measurement of shooting deviation

The center point of the circular display range on the monitor was represented by a black dot. The distance between the development of the iron nail on display and the center point of the display range was measured to evaluate the shooting deviation. The smaller the distance, the smaller the shooting deviation. If the distance between the two was 3 cm, the named shooting deviation was 3 cm, and if no nail was captured, the registered shooting deviation was the radius of the circular developing range of the display (14.2 cm). This study stipulated that when the shooting deviation was less than or equal to 3 cm, the shooting was qualified and stopped (a previous study by the research group found that the images taken when the shooting deviation was less than or equal to 3 cm could well meet the requirements of surgery). A bolt was placed on one side of the center point of the circular plate, and a nut was placed on the other side as a reference for shooting in the offset direction. The displayed picture was consistent with the position of the object so that the position of the C-arm could be adjusted by judging the shooting offset direction when simulating real work.

### Initial position of the C-arm machine and its positional relationship with the operating bed

The landmark warning tape was used to stick to the ground to fix the initial position of the C-arm, and the image intensifier of the C-arm was adjusted so that it was directly above the x-ray emitter. The plane of the image intensifier and the plane of the circular plate on the operating bed were adjusted so that the angle between them was Y°. When Y = 0°, both planes were in a horizontal state. The vertical distance from the center point of the image intensifier plane to the original plate plane was set as X cm. The values of X and Y were set according to the clinical experience values of high frequency used in routine intraoperative radiographs. The longitudinal axis of the C-arm machine (the C-arm machine console was located in the south, and the C-arm was located in the north) was perpendicular to the longitudinal axis of the operating table (the head of the bed was in the west, and the end of the bed was in the east). The initial position of the C-arm image intensifier was 50 cm from the northeast above the circular plate.

### Placement of circular plates and surgical beds

The photographic object used in this study was a round wooden board with a diameter of 38 cm and an iron nail with a diameter of 1.5 mm fixed at the center point. The round board was placed flat at the end of the operating bed, and the bed surface was kept horizontal. No obstacles were present under the bed so that the C-arm machine x-ray emitter could enter and exit freely. A surgical drape was laid on the operating table, and the lower edge was 40 cm off the ground to block the x-ray emitter of the C-arm machine. The aim was to prevent the photographer from judging the shooting position according to the position of the x-ray emitter of the C-arm machine.

### Laser light installation and debugging

The installation and debugging of the laser light must meet the following requirements: when the center of the circle surrounded by 10 laser points overlaps with the center of the circular board, the shooting result is that the development of the iron nails overlaps with the center point of the circular display range on the monitor.

### Location of Lead Shied, C-arm foot switch, and monitor

**Figure 7:**
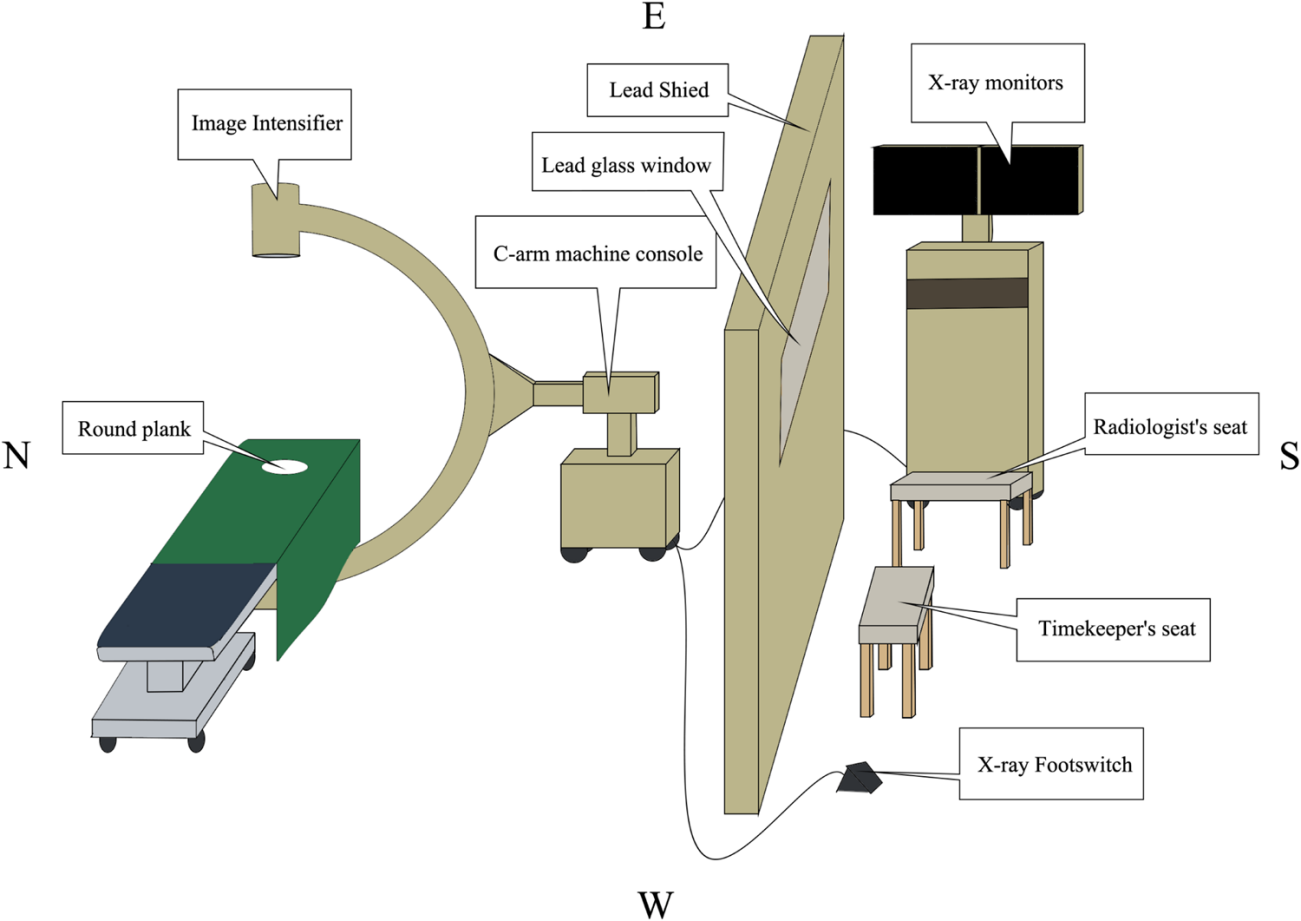
Schematic diagram of the layout of the lead shield, the X-ray foot switch, the monitor, the radiologist's seat, and the timekeeper's seat.

The lead shield was located 3 m south of the operating table, placed in an east–west direction, and parallel to the side of the operating table. The foot switch of the C-arm machine was placed on the west side behind the screen. The C-arm monitor was placed on the southeast side of the screen, and the display screen faced west. The aforementioned locations had landmarks for easy homing (Fig. 7).

### Seats for diagnostic radiologists and timekeepers

A diagnostic imaging physician sat on a stool in front of the monitor behind the screen, facing the monitor to assess shooting deviations at any time. A timekeeper, sitting on a stool in the middle behind the screen, watched the shooting scene through the lead glass. The aforementioned positions were marked on the ground for easy homing (Fig. 7).

### Training of the photographer

A unified training was conducted for photographers. The photographers were informed of the clinical significance and experimental precautions of this study. They were allowed to perform the shooting operation according to the experimental process and could judge the offset direction of the C-arm machine according to the shooting deviation.

### Photographer selection and group

**Figure 8:**
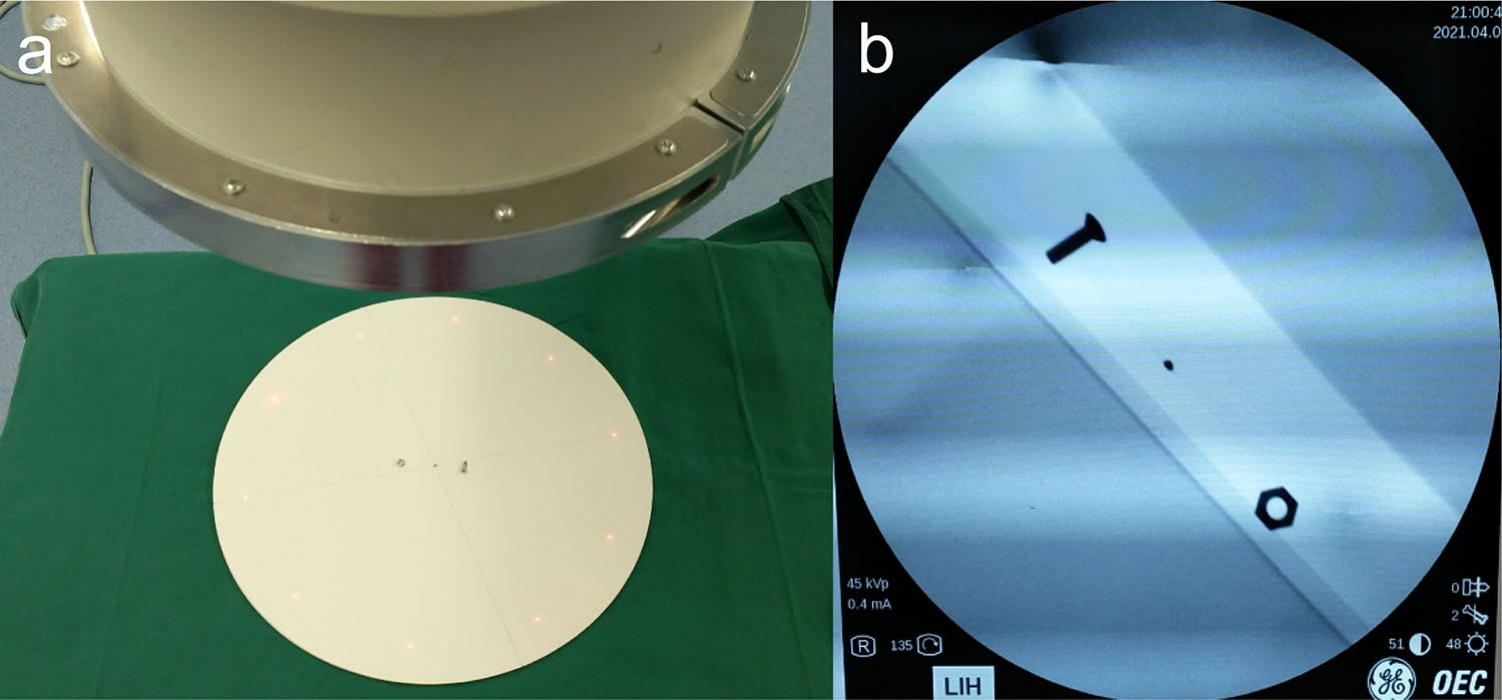
(**a**) C-arm camera with the locator was used to shoot the circular plate (*X* = 30 cm, *Y* = 0°). (**b**) Center nail of the display circular plate overlapped the center point of the display.

**Figure 9:**
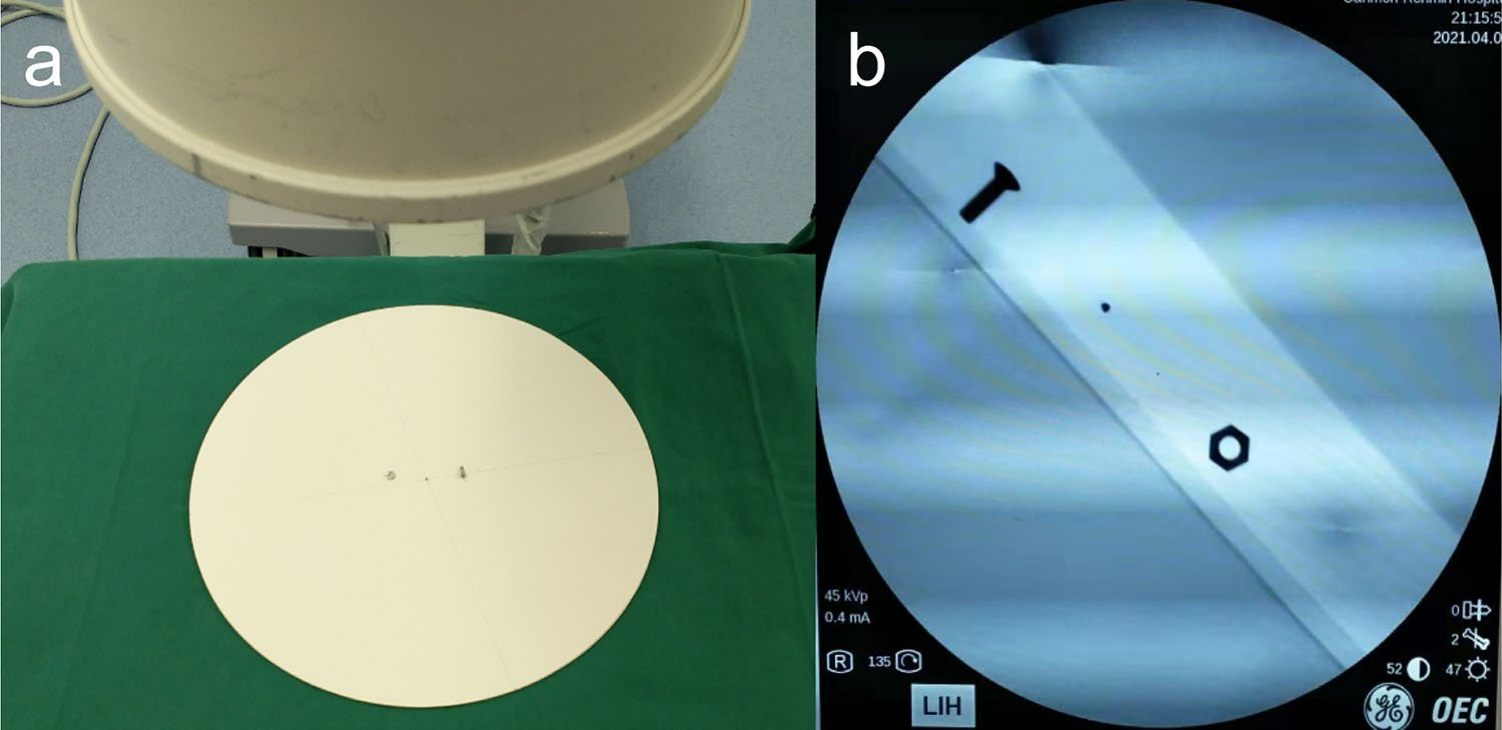
(**a**) C-arm camera without the locator shot the circular plate (*X* = 30 cm, *Y* = 0°). (**b**) Positional relationship between the center nail of the display circular plate and the center point of the display.

**Figure 10:**
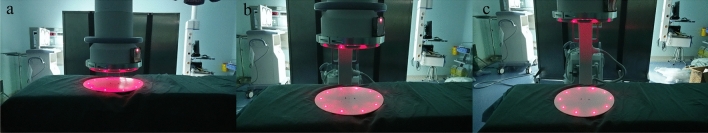
Three shooting distances (*X*) in the experimental group. (**a**) *X* = 10 cm. (**b**) *X* = 30 cm. (**c**) *X* = 50 cm.

**Figure 11:**
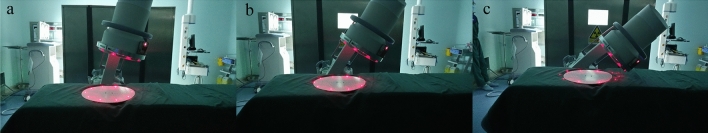
Three shooting angles (*Y*) in the experimental group. (**a**) *Y* = 15°. (**b**) *Y* = 30°. (**c**) *Y* = 45°.

The intraoperative photographers in primary hospitals are usually surgical doctors or operating room nurses, rather than professional radiology technologists. Hence, a total of 60 physicians and nurses from the surgical system of the hospital where the research group was located were randomly selected as the shooting operators. All of them operated two types of instruments: C-arms with self-made locators and C-arms without self-made locators. The group using the C-arm machine with the self-made locator to shoot the center point of the circular plate was the experimental group, and the group using the C-arm machine without the locator to shoot the center point of the circular plate was the control group. The shooting modes of the experimental group and the control group are shown in Figs. 8 and 9, respectively. The three shooting heights and shooting angles of the experimental group are shown in Figs. 10, 11, and Table 1, respectively.

**Table 1:** Six shooting modes.

Shooting mode	*X* (cm)	*Y*(°)
1	10	0
2	30	0
3	50	0
4	30	15
5	30	30
6	30	45

### Statistical analysis

SPSS 25.0 (IBM, NY, USA) software was used for statistically analyzing the number of shots, the total shooting time, and the first-shot deviation. *Kolmogorov–Smirnov* tests were used to test whether our data conformed to a normally distribution. *ANOVA* and *t*-test were used for normality distributed of the data, *Nonparametric* test is used for non-normally distributed data. *ANOVA* was used to compare between three different shooting conditions, *t*-test was used to compare the total shooting time of the two different shooting patterns, *Nonparametric* tests were used to compare the number of shots and first shot deviations for two different shooting patterns. *P* value < 0.05 indicated a statistically significant difference.

## Data Availability

The datasets generated during and/or analysed during the current study are available from the corresponding author on reasonable request.
